# What the “Voices” of Dysphagia Are Thinking about Its Future

**DOI:** 10.1055/s-0046-1822811

**Published:** 2026-06-02

**Authors:** Geraldo Pereira Jotz, Arthur Viana Jotz, Silvia Dornelles, Leandro Castro Velasco, Luciana Fernandes Costa, Luciana Miwa Nita Watanabe, Thais Gonçalves Pinheiro Vilela, Eliézia Helena de Lima Alvarenga, Evaldo Dacheux de Macedo Filho, Dayse Manrique, Vanessa Santos Elias, Lucia Mourão, Ana Maria Furkim, Natasha Marcarenhas Andrade Braga, Roberta Gonçalves da Silva, Rosane Sampaio Santos, Elisabete Carrara de Angelis

**Affiliations:** 1 Universidade Federal do Rio Grande do Sul (UFRGS), Porto Alegre, RS, Brazil; 2 Universidade do Vale do Rio dos Sinos (UNISINOS), Porto Alegre, RS, Brazil; 3 Hospital de Clínicas de Porto Alegre (HCPA), Universidade Federal do Rio Grande do Sul (UFRGS), Porto Alegre, RS, Brazil; 4 Instituto da Voz, Porto Alegre, RS, Brazil; 5 Hospital das Clínicas da Faculdade de Medicina da Universidade de São Paulo (HC-FMUSP), São Paulo, SP, Brazil; 6 Hospital Universitário de Brasília (HUB), Universidade de Brasília (UNB), Brasília, DF, Brazil; 7 Hospital Sírio Libanês – Brasília (HSL-Brasília), Brasília, DF, Brazil; 8 Hospital São Paulo, Escola Paulista de Medicina, Universidade Federal de São Paulo (UNIFESP), São Paulo, SP, Brazil; 9 Hospital Instituto Paranaense de Otorrinolaringologia (IPO), Curitiba, PR, Brazil; 10 Hospital Ortopédico Associação de Assistência à Criança Deficiente (AACD), São Paulo, SP, Brazil; 11 Clínica Qualos, Porto Alegre, RS, Brazil; 12 Universidade Estadual de Campinas (UNICAMP), Campinas, SP, Brazil; 13 Universidade Federal de Santa Catarina (UFSC), Florianópolis, SC, Brazil; 14 Universidade Federal da Bahia (UFBA), Salvador, BA, Brazil; 15 Universidade Estadual Paulista (UNESP – Marília), Marília, SP, Brazil; 16 Universidade Tuiuti do Paraná (UTIP), Curitiba, PR, Brazil; 17 Speech Therapy Department, Hospital A. C. Camargo, São Paulo, SP, Brazil

## Historical Aspects (Past, Present, and Future)

Over the past 3 decades, physicians, speech-language pathologist, professors, and researchers from diverse fields, such as physics, design, software engineering, and programming have dedicated themselves to providing healthcare to patients with dysphagia, driving significant advances in the understanding, diagnosis, and treatment of this condition. This movement has consolidated dysphagia as a highly-specialized and intrinsically-interdisciplinary field.

The historical evolution shows a transition from predominantly clinical and descriptive approaches to increasingly objective, quantitative, and technologically oriented diagnostic methods. These instruments allow for the systematic identification of patterns, favoring the early detection of impairments, and enabling the prediction of complications, particularly after acute neurological events or throughout the course of neurodegenerative diseases, among other clinical conditions.

Currently, there is a substantial deepening in the knowledge of the neurophysiology of normal swallowing, allowing for the characterization of different dysphagia phenotypes associated with specific clinical conditions, such as neurodegenerative diseases and healthy aging. The literature highlights contributions to a better and more refined understanding of the temporal measure of swallowing events and pharyngeal residue patterns, expanding the capacity for clinical interpretation and functional stratification of patients. 1

New technologies, such as flexible cervical devices, enable continuous, real-time monitoring of laryngeal movements and swallowing patterns outside the clinical setting, expanding care possibilities and promoting telemedicine.

The use of three-dimensional (3D) printing to create personalized foods with modified textures that are safe, nutritious, and appealing has been increasing, with research still in the experimental phase focusing on modulating food viscosity using hydrocolloids, starches, and proteins to create gels and emulsions in consistencies customized to each patient's needs. Furthermore, four-dimensional (4D) printing uses smart materials that change their configuration over time in response to external stimuli, such as temperature, pH, and humidity. In the area of dysphagia, treatment can be even more personalized, with medications and foods that can expand, bend, or deform after administration.

Narrowband imaging offers high resolution, and it is contributing to the refinement of diagnostic accuracy. However, the health field continues to conduct research from a regenerative perspective for the treatment of swallowing disorders.

Telehealth applications and platforms are emerging to assist in daily monitoring and therapy, allowing physicians to manage patients remotely with great efficiency.

Despite the rapid advancement in the treatment of patients with dysphagia globally, there is still a need to improve clinical practice. The integration of scientific approaches with artificial intelligence (AI) tools has the potential to promote a deeper understanding of the physiology and neurophysiology of swallowing, assisting in the standardization of assessment methods, a step prior to treatment. For the implementation of more effective practices, it is necessary to develop consensus guidelines that establish standardized parameters for clinical and research reports. This step is essential for the development of personalized evidence-based algorithms and the definition of robust metrics to measure therapeutic efficacy.

Medical trends with the potential to impact research and clinical practice for patients with dysphagia include, among others, advances in video endoscopy of swallowing, diagnostic imaging, personalized medicine, regenerative medicine, and telehealth. 2

In the present editorial, we will analyze the current state of research, diagnostic evaluation, and treatment of dysphagia, as well as make predictions about the future, highlighting some relevant aspects among the many that involve the topic.

## Innovations in the Diagnostic Field

Numerous innovations have emerged in the field of dysphagia, both in its diagnosis and monitoring. Despite this, these new tools need to be validated through well-designed studies and the clinical practice of professionals already adapted to conservative methods.

Among the main innovations in the diagnostic area, pharyngeal high-resolution manometry/pharyngeal high-resolution impedance manometry (P-HRM/P-HRIM) stands out. Unlike conventional manometry, P-HRM uses solid-state sensors (greater sensitivity) with reduced spacing (< 1 cm), allowing for visualization of the pharyngoesophageal transition without motion artifacts and providing detailed biomechanical information on the physiology of pharyngeal swallowing. As a positive factor, it allows for the objective quantification of intrabolus pressure and relaxation of the upper esophageal sphincter (UES), accurately differentiating between failures in pharyngeal propulsion and restriction of UES opening. The international consensus established by the P-HRM Working Group proposed the standardization of essential metrics, including the integrated relaxation pressure of the upper esophageal sphincter (UES-IRP) and intrabolus-hypopharyngeal pressures, providing objective parameters for evaluation. This standardization enables a much more precise and detailed phenotyping of UES dysfunctions, overcoming the limitations of conventional manometry and allowing for more accurate diagnoses that guide specific therapeutic decisions. 3 4

Another important innovation is the Dynamic Imaging Grade of Swallowing Toxicity for Fiberoptic Endoscopic Evaluation of Swallowing (DIGEST-FEES) scale, which enables the standardization of videoendoscopy of swallowing (VED/FEES) through a validated scale that mimics the rigor of videofluoroscopy (VFS). It presents high interobserver reliability for grading the severity of dysphagia based on two domains: safety (penetration/aspiration) and efficiency (residue). The study that validated this scale demonstrated a robust correlation between endoscopic findings and clinical outcomes. 5 Other scales are being validated as methods for standardizing dysphagia analysis using FEES.

The introduction of VFS in 3D introduces quantitative measures and attempts international validation for standardizing what is considered a swallowing disorder and its mapping.

The use of AI in video analysis, supported by machine learning techniques and, more specifically, deep learning models, although still in the implementation phase, already shows accuracy comparable to experts in detecting aspiration, including cases of silent aspiration. Recent studies demonstrate that deep learning algorithms using convolutional neural networks (CNNs) achieve an area under the curve (AUC) greater than 90% in aspiration detection, approaching the performance of experienced radiologists and otolaryngologists. This study demonstrated that AI could reduce interobserver variability in high-volume services by automating the calculation of Penetration-Aspiration Scale (PAS). 6 This advancement shows a paradigmatic shift in dysphagia, characterized by the incorporation of digital, noninvasive, and data-driven methods into clinical practice.

Wearable sensors represent a significant advancement in dysphagia diagnosis by enabling continuous and noninvasive recording of biomechanical and electrophysiological swallowing signals in real-world settings, overcoming the limitations of traditional tests based on point-in-time assessments. However, despite their great potential, this technology is still in the development and clinical validation phase, requiring robust studies and standardized protocols before its widespread incorporation into clinical practice.

There is still a need for further search into the standardization of FEES and its parameters in order to facilitate the clinical diagnosis of dysphagia, even in those patients without a diagnosis of the underlying disease (introduction and validation of a phenotypic classification of neurogenic dysphagia based on VED/FEES). Dysphagia phenotypes can facilitate differential diagnosis in patients with dysphagia of unclear etiology. 7

Regarding gastroesophageal reflux disease (GERD), we must highlight esophageal impedance-pH monitoring, which allows the physician to assess the reflux of acidic and nonacidic stomach contents into the esophagus by monitoring the patient for approximately 24 hours. This test is considered the most sensitive and specific for diagnosing GERD.

## Innovations in the Therapeutic Field

In recent decades, the treatment of dysphagia has improved rapidly thanks to research and clinical advances. However, diagnostic and therapeutic tools are far from perfect, as the swallowing system is complex, the needs of individual patients vary, and practices are still very diverse. The good news is that AI, big data science, robotics, and genetic profiling will revolutionize how we understand and treat dysphagia. And some of these advances will arrive sooner than you think. 8

Therapeutic advances in dysphagia reflect the increasing incorporation of concepts of neuroplasticity, muscle and behavioral training, and personalized therapy, significantly expanding the repertoire of available interventions and directing clinical practice towards more individualized approaches based on neurophysiological mechanisms.

Regarding innovations in the therapeutic area, we have pharyngeal electrical stimulation (PES), which, unlike transcutaneous neuromuscular stimulation (TNMES), uses an intraluminal catheter to directly stimulate the afferent nerves of the pharyngeal mucosa. This stimulation accelerates decannulation in tracheostomized patients after a stroke, reducing PAS results. The conclusion of this study was that PES significantly increased the readiness rate, favoring functional efficiency and helping to reduce the time required for decannulation of poststroke tracheostomized patients, with a robust safety profile. 9

Pharyngeal electrical stimulation has emerged as a promising approach, particularly in patients with neurogenic dysphagia following stroke. However, the literature still highlights controversies regarding its indiscriminate use.

In the field of neuromodulation, noninvasive brain stimulation has emerged as an innovative strategy. Techniques such as transcranial magnetic stimulation (TMS) and transcranial direct current stimulation (tDCS) have been investigated with the aim of modulating the cortical excitability of areas involved in swallowing control. Systematic reviews and meta-analyses demonstrate the effectiveness of neuromodulation in dysphagic patients due to strokes.

Furthermore, we have expiratory muscle strength training (EMST), which uses linear pressure load devices to strengthen the suprahyoid muscles through activation during forced expiration against resistance. This is observed in the increased anterior displacement of the hyoid bone and improved airway protection in patients with Parkinson's disease. This study demonstrated that four-week expiratory muscle strength training significantly reduces overall dysphagia severity in PD patients, with a sustained effect after 3 months compared with sham training. This was mainly achieved by improving swallowing efficiency. The treatment effect is probably caused by peripheral mechanisms, as no changes in the cortical swallowing network were identified. 10

One of the main challenges in translating scientific research findings into clinical practice lies in the variability in the definition of dysphagia − which can encompass swallowing safety, efficacy, or both − associated with the great phenotypic diversity of related clinical conditions. In this context, ongoing research lacks greater standardization, systematic evaluation of the reliability of classifications, and comparison of the diagnostic accuracy of VFS swallowing study and fiber-optic endoscopic swallowing assessment, essential elements for the consolidation of consistent, evidence-based clinical guidelines. Translational research has demonstrated the potential to transform future management strategies for head and neck cancer, directing them towards precision medicine, while increasing evidence emerges regarding the quantitative assessment of dysphagia associated with the treatment of this condition. Evidence is accumulating to support the effectiveness of strength training treatment protocols. A critical review of the existing literature highlights methodological issues that require further investigation regarding the application of noninvasive brain stimulation in dysphagic patients. 11

The field of dysphagia rehabilitation for head and neck cancer patients is evolving towards precision medicine. Future efforts should focus on developing personalized rehabilitation strategies based on individual risk stratification, integrating technology for monitoring and motivation, and promoting interdisciplinary collaboration among physicians, speech-language pathologists, and behavioral scientists to optimize efforts towards more lasting functional recovery. 12

Another area that is growing in studies and in clinical practice is prehabilitation. Prehabilitation in dysphagia refers to a set of interventions aimed at preparing patients with swallowing difficulties for surgical procedures or cancer treatments. The focus is on improving swallowing function and minimizing associated complications, such as aspiration and malnutrition. Through careful assessments, specific muscle strengthening exercises, nutritional guidance, and dietary modification techniques, prehabilitation seeks to optimize the patient's overall condition before treatment, promoting faster and more efficient recovery. This multidisciplinary approach is essential to improve the quality of life of individuals affected by dysphagia.

Still within the area of the challenging dysphagia after radiotherapy, studies with survivors of head and neck cancer have demonstrated what is called *very late dysphagia* ,” the worsening of dysphagic symptoms from the second decade postirradiation, especially when the patient is not followed by a multidisciplinary team, highlighting the importance of early follow-up.

A study that assessed the clustering of symptoms of dysphagic phenotypes in elderly patients through unsupervised machine learning using multidimensional features identified different dysphagia phenotypes based on multidimensional patient characteristics, with unique clinical features and outcomes. The results support the development of targeted therapeutic strategies to improve patient prognosis. 13

In the field of rehabilitation of oropharyngeal dysphagia of neurological origin, it is observed that, over the last four decades, the evolution of conventional techniques, including isolated and/or functional myotherapeutic exercises, behavioral approaches, and adjuvant therapies aimed at potentiating their effects, seems to have reached a therapeutic plateau. Given this scenario, the following question arises: how to approach the most refractory cases of dysphagia?

It is important to highlight that swallowing is not limited to the action of the muscles responsible for transporting the food bolus from the oral cavity to the stomach. It is a highly complex process, regulated by sophisticated neurological mechanisms that operate synchronously through elaborate polysynaptic circuits, also integrating social and affective dimensions related to the act of eating.

Additionally, future perspectives may include the development of technologies capable of promoting electrical release or neuroactive substances directly in brain tissue, as well as advances in stem cell research, expanding the range of therapeutic interventions for these refractory cases.

Assistive technologies equipped with devices that promote biofeedback, a key component of motor (re)learning, have become progressively more accessible, both in terms of availability and cost.

## Final Considerations

Dysphagia is increasingly prevalent in the population as global aging intensifies, and it is significantly associated with morbidity and mortality, with important socioeconomic implications.

The heterogeneity of patients and clinical conditions represents a challenge for translational medicine in dysphagia. Although there is a growing volume of research in the area, the incorporation of these findings into clinical practice is still limited, despite the emerging potential to support precision medicine in the near future.

Through precision medicine, researchers aim to develop diagnostic and therapeutic approaches that consider individual characteristics, including genetic factors, comorbidities, environment, and lifestyle. This approach integrates traditionally used clinical data, such as signs, symptoms, personal and family history, and complementary tests, with individual biological profiles.

By considering these multiple aspects, it becomes possible to move towards a more integrated and personalized approach, capable of optimizing the diagnosis and treatment of dysphagia. However, for this potential to materialize, it will be essential to invest in the standardization of methods, the rigorous validation of new technologies, and the continuous training of healthcare professionals. Only then will it be possible to ensure that scientific advances translate into real improvements in clinical outcomes and the provision of safer, more effective, and patient-centered care.
